# Relationship between autonomic cardiovascular control, case definition, clinical symptoms, and functional disability in adolescent chronic fatigue syndrome: an exploratory study

**DOI:** 10.1186/1751-0759-7-5

**Published:** 2013-02-07

**Authors:** Vegard B Wyller, Ingrid B Helland

**Affiliations:** 1 Department of Paediatrics, Oslo University Hospital and University of Oslo, Oslo, Norway; 2 Department of Paediatrics, Oslo University Hospital Rikshospitalet, N-0027, Oslo, Norway

**Keywords:** Adolescents, Chronic fatigue syndrome, Autonomic cardiovascular control, Diagnostic criteria

## Abstract

Chronic Fatigue Syndrome (CFS) is characterized by severe impairment and multiple symptoms. Autonomic dysregulation has been demonstrated in several studies. We aimed at exploring the relationship between indices of autonomic cardiovascular control, the case definition from Centers for Disease Control and Prevention (CDC criteria), important clinical symptoms, and disability in adolescent chronic fatigue syndrome. 38 CFS patients aged 12–18 years were recruited according to a wide case definition (ie. not requiring accompanying symptoms) and subjected to head-up tilt test (HUT) and a questionnaire. The relationships between variables were explored with multiple linear regression analyses. In the final models, disability was positively associated with symptoms of cognitive impairments (p<0.001), hypersensitivity (p<0.001), fatigue (p=0.003) and age (p=0.007). Symptoms of cognitive impairments were associated with age (p=0.002), heart rate (HR) at baseline (p=0.01), and HR response during HUT (p=0.02). Hypersensitivity was associated with HR response during HUT (p=0.001), high-frequency variability of heart rate (HF-RRI) at baseline (p=0.05), and adherence to the CDC criteria (p=0.005). Fatigue was associated with gender (p=0.007) and adherence to the CDC criteria (p=0.04). In conclusion, a) The disability of CFS patients is not only related to fatigue but to other symptoms as well; b) Altered cardiovascular autonomic control is associated with certain symptoms; c) The CDC criteria are poorly associated with disability, symptoms, and indices of altered autonomic nervous activity.

## Background

Chronic fatigue syndrome (CFS) is a disabling condition that seriously affects school-attendance and social activities [1]. The prevalence among 8–17 years olds has been reported as high as 1% [2,3]; thus, CFS constitutes a substantial health problem in adolescence. As yet, no effective pharmacotherapy exists.

CFS patients report a great variety of bodily symptoms. Generally, their experience of overwhelming fatigue is assumed to be the single most important factor for their substantial impairments. However, it is conceivable that other symptoms might equally impact on their abilities. If so, this might be important for clinical management and suggest alternative treatment strategies. The relationship between symptoms and disability has rarely been explored in previous CFS reports.

Certain characteristic CFS symptoms, such as lightheadedness, indicate alterations of autonomic nervous activity. Indeed, experimental studies have demonstrated distinct abnormalities in cardiovascular autonomic control [4,5]. Previous reports from our institution have documented higher blood pressure and heart rate at rest among adolescent CFS patients as compared to healthy controls and a stronger increase of these variables upon orthostatic stress [6-10]. Similarly, ambulatory measurements of blood pressure and heart rate indicate higher nocturnal values among CFS patients as compared to controls [11]. Taken together, these studies suggest that abnormal autonomic nervous activity might constitute an important aspect of CFS pathophysiology [12,13]. If so, a relationship between clinical symptoms and indices of altered autonomic cardiovascular control should be demonstrable, which in turn might provide a basis for development of novel therapies as well as a diagnostic test.

As yet, no CFS biomarker has any diagnostic utility; thus, the diagnosis is solely based upon patients’ reports of symptoms. Several different case definitions exist, reflecting unsettled controversies in the scientific community [14,15]. The definition from the International Chronic Fatigue Syndrome Study Group at the Centers for Disease Control and Prevention (commonly referred to as the CDC-definition) appears to be most frequently used [16]. This definition requires at least six months of unexplained chronic or relapsing fatigue of new onset, severely affecting daily activities, as well as four or more of eight specific accompanying symptoms (headache, muscle pain, joint pain, sore throat, tender lymph nodes, impaired memory or concentration, unrefreshing sleep, and malaise after exertion). The validity of this definition has been questioned [17-20]. For instance, a formal factor analysis of symptoms in a broadly defined group of chronic fatigued patients did not show a strong correspondence with the CDC accompanying symptoms [19]. Accordingly, a recent study by Sullivan and co-workers based upon the Swedish twin registry concluded that there was no empirical support for the requirement of four out of eight CDC accompanying symptoms [20]. In adolescents, few studies of validity have been conducted. Thus, one purpose of this study is to explore the informative value of the CDC-definition within a more broadly defined population of adolescent chronic fatigue sufferers; this approach has been recommended by others [20,21].

In 2007, we launched an observational prospective study on the clinical course of adolescent CFS. The changes of different clinical features with time have been reported elsewhere [22]. However, little is known about the interrelation of pathophysiological characteristics, patients’ experiences, and functional abilities. Thus, the overall aim of the present report is to explore the relationship between indices of autonomic cardiovascular control, the CDC case definition, important clinical symptoms, and disability in adolescent chronic fatigue syndrome at a single time point.

## Methods

### Subjects

During the study period from August 2007 to April 2009, adolescent CFS patients aged 12 – 18 years were recruited from the Paediatric outpatient clinic, Oslo University Hospital, Rikshospitalet, Norway, which serves as a national referral center for children and adolescents with CFS. Prior to referral, they had been tentatively given the diagnosis CFS at local hospitals. The Norwegian clinical guidelines for diagnosis of adolescent CFS are in accordance with the recommendations from The Royal College of Paediatrics and Child Health [1] and the National Institute for Health and Clinical Excellence [23]. In short, a diagnosis of CFS is made if the patients suffer from three or more consecutive months of unexplained disabling fatigue worsened by physical or mental exertion. No accompanying symptoms are required.

At our referral centre, diagnostic confirmation was established by means of a thorough and standardized set of investigations, ruling out differential diagnosis such as autoimmune, endocrine, neurologic or psychiatric disorders (including depression). All patients included in this study fulfilled our clinical definition of CFS. In addition, inclusion in this study required no other chronic disorders, no permanent use of pharmaceuticals, and no current demanding life event that might explain the fatigue.

Thus, the inclusion criteria in this study are wider than the CDC criteria. This approach is in line with findings from recent validation studies [19,20], and has also been shown to be feasible in previous studies from our institution [6-11]. Subgrouping of the participants according to the CDC case definition was performed post hoc, based on questionnaire results (cf. below). The main reason for not adhering to the CDC case definition was too few accompanying symptoms.

### Study protocol

CFS patients were invited to an experimental session (consisting of head-up tilt-test (HUT) and a questionnaire) at our institution. One week prior to the experiments, all participants were instructed not to drink beverages containing alcohol or caffeine, not to take any drugs, and not to use tobacco products. Patients were summoned for a second visit 3–17 months after the first experimental session; the results from this follow-up visit have been reported elsewhere [22]. Written, informed consent was obtained from all participants and their parents. The study was conducted in accordance with the Declaration of Helsinki and was approved by the Regional committee for ethics in medical research.

### Questionnaire

The Fatigue Severity Scale (FSS) [24], which is a validated instrument for assessing fatigue, was translated into Norwegian by one of the authors (VBW) and slightly modified in order to fit our particular age group. The modified FSS consists of 7 items having a 1–5 Likert scale (agree/disagree). Examples of single items are: “I get exhausted from physical activity” and “Fatigue is among my three most troublesome symptoms.”

In addition, the questionnaire consisted of items addressing the frequency of a wide range of common CFS symptoms (including the accompanying symptoms of the CDC case definition) and impairments (such as school absenteeism) on 1–5 Likert scales. These questions were based upon our clinical experience and have also proven feasible in previous studies from our institution [7,9].

### Head-up tilt-test (HUT)

HUT was undertaken according to a revised protocol as described elsewhere [7]. The feasibility of this protocol for studying adolescent CFS patients has been demonstrated in several previous studies [6-8]. In particular, the low tilt angle (20°) does not normally precipitate syncope, which is otherwise a common problem among adolescents being subjected to stronger orthostatic challenges [25]. Still, 20° head-up tilt is sufficient to demonstrate hemodynamic alterations among CFS patients compared to healthy controls.

HUT was performed at daytime between 8 a.m. and 3 p.m. under calm conditions, dimmed lights, and a stable room-temperature of 23 - 25°C. Patients were attached to the Task Force Monitor® (Model 3040i, CNSystems Medizintechnic, Graz, Austria); a combined hardware and software device for noninvasive continuous recording of cardiovascular variables [26]. They were positioned horizontally on a tilt-table with foot-board support (Model 900–00, CNS-systems Medizintechnik, Graz, Austria) and had a safety strap over the waist. After 5 minutes of baseline recordings, they were head-up tilted 20° for 15 minutes, followed by another 5 minutes epoch in the horizontal position. Subjects did not speak and were not spoken to.

Instantaneous heart rate (HR) was obtained from the R-R interval (RRI) of the electrocardiogram. Photoplethysmography on the right middle finger was used to obtain a non-invasive, continuous recording of arterial blood pressure. This method correlates satisfactorily with invasive pressure measurements [27] and has been validated in adolescents and children [28]. Impedance cardiography was used to obtain a continuous recording of the temporal derivate of the transthoracic impedance (dZ/dt) [29]; the impedance recordings are not reported in this article.

All recorded signals were on-line transferred to the built-in recording computer of the Task Force Monitor® that was running software for real-time data acquisition. RRI was subjected to spectral analyses using an adaptive autoregressive algorithm for calculating spectral power densities in the low-frequency (LF) (0.04 – 0.15 Hz) and high-frequency (HF) ranges (0.15 – 0.4 Hz). HF-RRI is considered an index of parasympathetic sinus node modulation, whereas LF-RRI is related to the combined influence of sympathetic and parasympathetic nervous activity on the sinus node [26]. Beat-to-beat mean arterial blood pressure (MBP) was calculated by numerical integration of the recorded instantaneous blood pressure signal.

### Data analysis

Data were exported to Microsoft Excel for further analyses. Individual items from the questionnaire that addressed clinical symptoms were sorted and labeled based upon assumptions about underlying mechanisms; for instance, items regarding problems of memory and problems of concentration were both assumed to address cognitive alterations in CFS [7,9]. The arithmetical mean was calculated for variables consisting of more than one item. Likewise, a Fatigue severity score was created by taking the arithmetical mean across all 7 items from FSS [24]. Finally, a disability score was defined as the arithmetical mean across 8 items addressing different aspects of impairment and malfunctioning: School attendance, homework, school physical activity, leisure activity, being with friends, being with family, going outdoors, and getting up from bed.

From each experimental run of HUT, we calculated the median of all variables in two epochs: From 270 to 30 seconds prior to tilt (Baseline) and from 300 to 540 seconds after being tilted (Tilt). We also computed delta values (Tilt – Baseline), which reflect the cardiovascular response to the tilt maneuver. This method of analyzing results from HUT has successfully been used in previous projects from our institution [6-8].

### Statistical analyses

**Figure 1:**
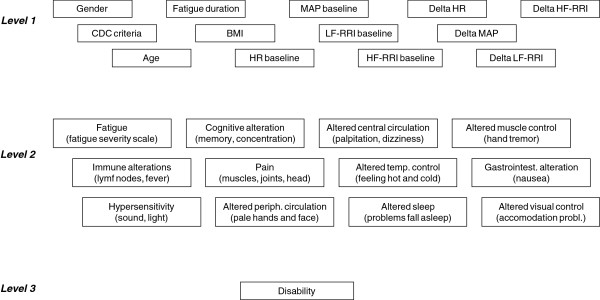
**Analytical model for the multivariate linear regression analyses.** We assumed that every variable on a “lower” level could be associated with variables “higher” up in the hierarchy.

Statistical analyses were performed using SPSS software (SPSS Inc., Chicago, Ill.). Based upon present evidence, we constructed an analytical model as outlined in Figure 1. We assumed that every variable on a “lower” level could be associated with variables “higher” up in the hierarchy. The potential relationship between variables was first explored in bivariate linear regression analyses, and then subjected to multivariate linear regression analyses. The selection of variables in the multivariate models was based upon results from bivariate analyses, their impact on the coefficients of the other variables, and their theoretical plausibility. In each multivariate model, the distribution of residuals was assessed for normality.

Results are reported as regression coefficients (Bs) with 95% confidence intervals (CIs). A p-value < 0.05 was considered statistically significant.

## Results and discussion

Initially, 47 CFS patients were included in the study. Of these, 38 patients (8 males, 30 females; mean age 15 years) completed the study protocol and were thus available to analyses (Table 1). The criteria of the CDC-definition were fulfilled by 27 patients (71%); background variables and variables reflecting autonomic cardiovascular control were similar in the subgroup that adhered and the subgroup that did not adhere to this definition (Table 1). Overall, in the patient group, the mean Fatigue severity score was 4.7 and thus close to the upper limit (scale from 1 to 5); in addition, patients had high scores in several other symptom domains.

**Table 1:** Variables subjected to linear regression analyses among CFS patients

	***CFS-patients***			Healthy controls^*^(n = 33)
	***Overall (n = 38)***	***CDC+ (n=27)***	***CDC– (n = 11)***	
*Level 1, background and cardiovascular*				
Female gender	30 (79 %)	23 (85 %)	7 (64 %)	19 (58 %)
	*Mean (CI)*	*Mean (CI)*		
Age (years)	15	15	15	15
	(14.4 to 15.5)	(14.2 to 15.7)	(14.2 to 16.0)	(14.5 to 15.8)
BMI (kg/m^2^)	22.3	22.3	22.2	20.6
	(20.8 to 23.8)	(20.7 to 23.9)	(18.5 to 26.0)	(19.7 to 21.5)
Fatigue duration (months)	24	25	23	
	(20 to 28)	(19 to 30)	(18 to 28)	
MAP baseline (mm Hg)^**^	82.2	83.1	79.8	80.0
	(79.4 to 84.9)	(79.9 to 86.4)	(73.8 to 85.8)	(77.8 to 82.3)
HR baseline (beats/min)^**^	72.7	73.7	70.4	66.8
	(68.9 to 76.6)	(68.9 to 78.5)	(63.0 to 77.7)	(63.2 to 70.4)
LF-RRI baseline (nu) ^**^	38.3	37.2	40.9	40.8
	(32.3 to 44.2)	(29.6 to 44.9)	(30.7 to 51.1)	(34.5 to 47.1)
HF-RRI baseline (nu) ^**^	61.7	62.8	59.1	59.2
	(55.8 to 67.7)	(55.1 to 70.4)	(48.9 to 69.3)	(52.9 to 65.5)
LF-RRI baseline (ms^2^) ^**^	600	478	899	963
	(331 to 868)	(280 to 677)	(29 to 1768)	(608 to 1318)
HF-RRI baseline (ms^2^) ^**^	1120	1038	1319	1859
	(700 to 1539)	(606 to 1470)	(186 to 2452)	(964 to 2753)
Delta MAP (mm Hg)^**^	5.4	4.3	7.8	−0.2
	(3.8 to 6.9)	(2.6 to 6.1)	(4.8 to 10.9)	(−1.4 to 1.0)
Delta HR (beats/min)^**^	4.0	4.7	2.3	1.0
	(2.3 to 5.7)	(2.5 to 6.8)	(−0.9 to 5.6)	(−0.3 to 2.3)
Delta LF-RRI (nu) ^**^	8.4	8.7	7.8	−1.2
	(3.8 to 13.1)	(2.6 to 14.7)	(0.3 to 15.4)	(−4.7 to 2.3)
Delta HF-RRI (nu) ^**^	−8.4	−8.7	−7.8	1.2
	(−13.1 to −3.8)	(−14.7 to −2.6)	(−15.4 to −0.3)	(−2.3 to 4.7)
Delta LF-RRI (ms^2^) ^**^	−127	−16	−398	−262
	(−329 to 76)	(−166 to 134)	(−1042 to 247)	(−446 to −77)
Delta HF-RRI (ms^2^) ^**^	−372	−266	−632	−456
	(−618 to −126)	(−458 to −74)	(−1412 to 147)	(−782 to −131)
*Level 2, symptoms (1–5 scale)*				
Fatigue^†^	4.7			
	(4.5 to 4.8)			
Cognitive alterations^‡^	3.1			
	(2.8 to 3.5)			
Altered temperature control	3.0			
	(2.6 to 3.4)			
Altered sleep	2.9			
	(2.3 to 3.4)			
Hypersensitivity^§^	2.7			
	(2.3 to 3.1)			
Pain^a^	2.6			
	(2.3 to 3.0)			
Altered peripheral circulation^b^	2.6			
	(2.2 to 3.1)			
Altered central circulation^c^	2.5			
	(2.2 to 2.9)			
Gastrointestinal alterations	2.4			
	(2.1 to 2.8)			
Altered visual control	2.2			
	(1.7 to 2.7)			
Immune alterations^d^	1.6			
	(1.3 to 1.8)			
Altered muscle control	1.6			
	(1.2 to 2.0)			
*Level 3, function (1–5 scale)*				
Disability^e^	2.9			
	(2.7 to 3.1)			

*Although not the aim of this article, reference values from healthy control subjects are displayed for clarity. Details concerning this reference material have been described elsewhere [7].**Obtained from 20^o^ head up tilt test. ^†^Fatigue severity scale [23]. ^‡^Mean of the following items: Difficult to concentrate; difficult to remember. ^§^Mean of: Sensitivity towards sounds; sensitivity towards light. ^a^Mean of: Headache; muscle pain; joint pain. ^b^Mean of: Pale hands and feet; pale face. ^c^Mean of: Upright dizziness; palpitations. ^d^Mean of: Enlarged cervical lymph nodes; sensation of fever. ^e^Mean of: School attendance; homework; school physical activity; leisure activity; being with friends; being with family; going outdoors; getting up from bed.

CI=confidence interval. CDC=Centers for disease control and prevention. CDC+ = adherence to CDC diagnostic criteria. CDC– = not adhering to CDC diagnostic criteria. BMI=body mass index. MAP=mean arterial pressure. HR=heart rate. LF-RRI=low-frequency variability of RR-interval (i.e. heart rate). HF-RRI=high-frequency variability of RR-interval (i.e. heart rate). n.u.=normalized units.

In order to avoid the methodological problem of multiple testing, no statistical tests have been performed.

**Figure 2:**
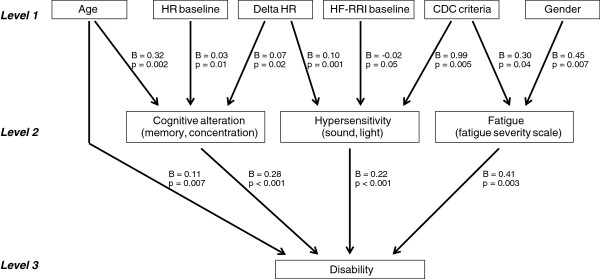
**Results from the final multivariate linear regression analyses.** B=linear regression coefficient (unstandardized).

In bivariate linear regression analyses, several variables at level 1 and level 2 were significantly related to disability. In multivariate analyses, however, only the relationships to cognitive alteration, hypersensitivity, fatigue (all level 2) and age (level 1) remained statistically significant (Table 2, Figure 2). Thus, a high disability score is independently related to cognitive symptoms, hypersensitivity symptoms, high fatigue score, and older age. The final model explained 77% of the variance in disability.

**Table 2:** The relationship between disability and variables at levels 1+2

	***B (CI)***	***p-value***
Cognitive alteration	0.28	< 0.001
	(0.16 to 0.41)	
Hypersensitivity	0.22	< 0.001
	(0.11 to 0.32)	
Fatigue	0.41	0.003
	(0.15 to 0.67)	
Age	0.11	0.007
	(0.03 to 0.18)	
	*R*^*2*^	
Disability variance explained from model	0.77	

Multivariate linear regression analysis, final model.

Cognitive alteration, in turn, was significantly related to HR baseline, Delta HR and age (Table 3, Figure 2). In other words, cognitive symptoms are independently associated with high HR at rest, enhanced HR response during orthostatic challenge, and older age. Furthermore, hypersensitivity was significantly related to Delta HR (ie, high hypersensitivity scores were associated with enhanced HR response), HF-RRI baseline (high hypersensitivity scores were associated with low HF-RRI), and adherence to the CDC criteria (Table 4). Fatigue was significantly related to gender (females tended to be more fatigued than males) and adherence to the CDC criteria (Table 5).

**Table 3:** The relationship between cognitive alteration and variables at level 1

	***B (CI)***	***p-value***
HR baseline	0.03	0.010
	(0.01 to 0.06)	
Delta HR	0.07	0.024
	(0.01 to 0.13)	
Age	0.32	0.002
	(0.13 to 0.51)	
	*R*^*2*^	
Cognitive alteration variance explained from model	0.36	

Multivariate linear regression analyses, final model.

**Table 4:** The relationship between hypersensitivity and variables at level 1

	***B (CI)***	***p-value***
Delta HR	0.10	0.001
	(0.05 to 0.16)	
Adherence to CDC criteria	0.99	0.005
	(0.3 to 1.7)	
HF-RRI baseline	−0.02	0.050
	(−0.03 to 0.00)	
	*R*^*2*^	
Hypersensitivity variance explained from model	0.50	

Multivariate linear regression analyses, final model.

**Table 5:** The relationship between fatigue and variables at level 1

	***B (CI)***	***p-value***
Gender	0.45	0.007
	(0.13 to 0.78)	
Adherence to CDC criteria	0.30	0.041
	(0.01 to 0.59)	
	*R*^*2*^	
Fatigue variance explained from model	0.32	

Multivariate linear regression analyses, final model.

Summing up, the most important results in this study are:

a) The disability of CFS patients is not only related to fatigue but to other symptoms as well.

b) Altered autonomic cardiovascular control is associated with certain symptoms.

c) The CDC definition is rather poorly associated with disability, clinical symptoms and indices of altered autonomic cardiovascular control in this broadly defined CFS population.

### Association between disability and other clinical symptoms

The multivariate analyses suggest that cognitive alteration, hypersensitivity, and fatigue are equally associated with disability (Figure 2, Table 2). Of note, the mean score for these symptoms were also quite high (Table 1).

It is generally acknowledged that an experience of cognitive impairments is an essential part of CFS. Neuropsychological tests have revealed attenuation of executive control function, suggesting involvement of prefrontal cortical regions [30]. Also, cognitive behavioral therapy (CBT) appears to be the best documented treatment strategy for CFS, in adults [31] as well as in adolescents [32], even though opponents of CBT tend to claim that the effect is overstated [33]. CBT is not designed to improve cognitive function *per se*; still, it is noteworthy that the results of this study suggest that improving cognitive functions might have an independent, positive effect upon patients’ functional level even when their experience of fatigue is unaltered.

Hypersensitivity towards sensory stimuli is considered a hallmark of CFS pathophysiology in a recently proposed model [34]. In the latest case definition proposal, this symptom is given less attention than several other disease manifestations, such as symptoms of immune alterations [35]. In this study, however, symptoms of immune alterations were among the least prevalent bodily complaints (Table 1), and were not related to disability in the multivariate regression analysis (Table 2).

Generally, it is interesting that most symptoms were associated with disability in bivariate analyses but did not remain significantly associated in multivariate analyses. Although different explanations are possible, this is congruent with theories suggesting that most CFS symptoms are manifestations of a single, underlying pathophysiological mechanism [12,34], and questions the validity of highly specified, multidimensional case definitions [35].

### Association between clinical symptoms and autonomic cardiovascular control

In this study, increased heart rate at supine rest (HR baseline) was related to symptoms of cognitive alterations (Table 3), exaggerated heart rate response during orthostatic challenge (Delta HR) was related to cognitive alterations, and hypersensitivity, and low heart rate variability in the high-frequency range at supine rest (HF-RRI baseline) was associated with hypersensitivity. This finding complies with previous reports of an association between orthostatic test results and clinical symptoms [36-39] and suggests that head-up tilt testing might become a valuable diagnostic tool in CFS; however, this would require validation in a larger patient population, combined with a refinement of test procedures. Also, the results indicate, in line with scattered case reports [40], that direct pharmaceutical treatment of cardiovascular autonomic alterations might improve clinical symptoms in CFS. This hypothesis should be tested in a randomized controlled trial. Furthermore, such treatment might be beneficial with regard to long-term cardiovascular health, as increased basal heart rate [41], as well as exaggerated cardiovascular responsiveness to challenges [42] are considered risk factors for cardiovascular morbidity.

Based upon our previous findings of cardiovascular autonomic alterations, we have proposed a ‘sustained arousal’-model of disease mechanisms in CFS [12]. This model suggests that hyperactivity of the brain stem *locus coeruleus* (LC) is a core phenomenon that promotes a sympathetic vs. parasympathetic shift in autonomic effector systems and thus explains increased heart rate at rest, low heart rate variability in the high-frequency range at rest, and exaggerated heart rate responses [43]. In relation to the present study, it is noteworthy that LC hyperactivity also attenuates cognitive abilities – in particular executive functions through the influence on prefrontal cortical areas [44]. Thus, the association between HR baseline/Delta HR and cognitive alterations, as documented in this study, seems to support the ‘sustaind arousal’-model. Also, LC activation promotes amplification of sensory afferent neurotransmission [45]; this effect in turn suggests a common ground for exaggerated heart rate response during tilt, which could be explained from enhanced baroreceptor input to the central reflex center [10] and symptoms of hypersensitivity.

### Association between adherence to the CDC definition and other variables

As the CDC criteria of CFS are based upon symptoms only, associations between adherence to these criteria and certain clinical manifestations follows directly from the definition. What is noteworthy in this study, though, is that these associations are rather weak (Figure 2). Furthermore, adherence to the CDC criteria is not independently associated with disability, and we did not find any association to HR baseline, HF-RRI baseline, nor Delta HR (Table 1). Thus, within a broadly defined population of CFS patients, the subgroup adhering to the CDC criteria is not characterized by a certain level of disability, nor is this subgroup specifically related to those indices of altered cardiovascular autonomic control that predicts clinical symptoms. The latter is in accordance with a previous study from our group, which failed to demonstrate a relation between autonomic dysregulation and psychosocial load in adolescent CFS [46].

These results, in concert with other findings suggesting that all chronic fatigue states share a relatively stereotyped set of symptoms [47], add to the concerns about the validity of the CDC definition, in particularly among adolescents [18-20]. Indeed, a large epidemiological survey suggests that CFS should be lumped together with several other ‘functional’ disorders, such as fibromyalgia and irritable bowel syndrome [48]. The controversies regarding diagnostic criteria constitute a great challenge for the CFS scientific community. One approach to clarify this issue in further studies would be to apply wider inclusion criteria than has been common so far, and thereafter perform subgroup analyses based on different case definitions [21].

### Study limitations

A strong limitation of this study is the limited number of participants, which particularly weakens the statistical power in multivariate analyses. Also, we have insufficient data regarding the participants that dropped out of the study, making the generalizability of the results more uncertain. The fatigue severity scale has a strong ceiling effect and was not normally distributed; this may have lead to underestimation of the associations involving this variable. The disability score has not been formally validated; although we consider the face validity to be reasonable, further studies in the field should use formally validated instruments, such as the Functional Disability Inventory [49]. As this was an exploratory study, we did not adjust the p-values for multiple statistical testing; it should be noted, however, that the majority of p-values were far beyond the level of significance and thus would probably have remained significant despite rigorous adjustments.

Of note, the documented associations between autonomic cardiovascular control, clinical symptoms, and disability are not proof of causality. Also, our three-level model should be regarded as a framework for explorative analyses only. Generally, the interactions might be far more complex than what we have shown here; investigating this possibility would require a larger number of participants and a prospective design allowing repeated measurements.

## Conclusions

In this exploratory study of adolescent CFS patients, disability was associated with cognitive alterations, hypersensitivity, and fatigue; cognitive alterations and hypersensitivity were associated with indices of altered cardiovascular autonomic control; and adherence to the CDC-criteria was poorly associated with other variables. These associations, which may have an important impact on clinical handling, should be further explored in a large-scale clinical study.

## Abbreviations

CFS: Chronic Fatigue Syndrome; CDC: Centers for Disease Control and Prevention; HUT: Head-up Tilt Test; HR: Heart Rate; MBP: Mean Arterial Blood Pressure; RRI: RR-interval; HF: high-frequency; LF: low-frequency.

## Competing interests

The authors declare no competing interests.

## Authors’ contributions

VBW conceived of the study, participated in data collection, carried out the statistical analyses, and drafted the manuscript. IBH participated in study design, data collection and analytic work, and helped to draft the manuscript. Both authors have read and approved the final version of the manuscript.
